# Serologic assays for the detection and strain identification of *Pteropine orthoreovirus*

**DOI:** 10.1038/emi.2016.35

**Published:** 2016-05-11

**Authors:** Harpal Singh, Masayuki Shimojima, Shuetsu Fukushi, Aiko Fukuma, Hideki Tani, Tomoki Yoshikawa, Satoshi Taniguchi, Ming Yang, Masami Sugamata, Shigeru Morikawa, Masayuki Saijo

**Affiliations:** 1 Department of Human Mechatronics System, Graduate School of Systems Design, Tokyo Metropolitan University, Tokyo 191-0065, Japan; 2 Department of Virology I, National Institute of Infectious Diseases, Tokyo 208-0011, Japan; 3 Department of Hygiene and Public Health, Graduate School of Human Health Sciences, Tokyo Metropolitan University, Tokyo 192-0397, Japan; 4 Department of Veterinary Science, National Institute Infectious Diseases, Tokyo 162-8640, Japan

**Keywords:** emerging infectious disease, prevention and control of communicable diseases, *pteropine orthoreovirus*, respiratory infections, serologic test

## Abstract

*Pteropine orthoreovirus* (PRV), potentially of bat origin, is reported to be a causative agent of emerging respiratory tract infections among humans in Southeast Asia. We evaluated the efficacy of serologic assays using the major outer capsid and cell attachment proteins (CAP) of PRV strains in the screening, confirmation and identification of three groups of human PRV infections; Indonesian/Japanese, Indonesian/Hong Kong and Malaysian strains. The different serologic assays were tested using rabbit polyclonal antisera raised against these proteins of selected PRV strains, and validation was carried out using sera from a Miyazaki-Bali/2007 PRV-infected patient and the patient's contacts. The results of this study showed that rabbit polyclonal antisera raised against the CAP of the Miyazaki-Bali/2007 PRV strain showed the highest reactivity to the Miyazaki-Bali/2007 PRV and to a lesser extent, cross-reactivity with the HK23629/07 and Melaka PRVs, respectively. Neutralization activity against the Miyazaki-Bali/2007 PRV was observed using rabbit anti-Miyazaki-Bali/2007 PRV CAP (320) but not with rabbit anti-HK23629/07 (<20) and Melaka (<20) PRV CAP. This lack of cross-neutralization, suggests the potential for human reinfection with different strains. The use of sera collected from contacts of the Miyazaki-Bali/2007 PRV-infected patient suggested that human-to-human infections with PRV are unlikely. Previously reported cases of PRV infections among human have been mild. However, the expanding geographic distribution of these viruses, of which its virulence remains unknown, warrants close monitoring to enable the development of prevention and control strategies in the event that a change in virulence occurs.

## INTRODUCTION

*Pteropine orthoreovirus* (PRV), which is potentially of bat origin, was previously reported as a novel cause of human respiratory tract infections that were centered in Southeast Asia, Malaysia^1, 2, 3, 4, 5^ and Indonesia, from which imported cases were subsequently reported in Japan^6^ and Hong Kong.^7, 8^ These human pathogenic strains, which show a phylogenetic relationship to orthoreoviruses of bat origin that have been isolated in Southeast Asia,^2, 9, 10^ China^11, 12^ and Australia,^2, 13, 14^ suggest that the contribution of various bat species to the spillover of the viruses to humans is causing novel and emerging PRV infections.^1, 2, 12^ In addition, a serological study on PRV infections in Central Vietnam revealed that 4.5% of the population has PRV antibodies, suggesting that PRV infections may be more prevalent than previously thought.^15^

The structural characteristics of PRV (that is, it is composed of 10 discrete gene segments) can facilitate genetic reassortment between PRV strains.^1, 2, 3, 4, 5, 6, 7, 8, 9, 10, 11, 12, 13, 14, 15, 16^ The potential for genetic reassortment is a major determinant of the evolution of PRV.^2^ Although the previously reported human cases have exhibited mild respiratory symptoms with limited human-to-human transmission,^1, 2, 3, 4, 5, 6, 7, 8^ the recent increase in reported cases highlights the need for the surveillance of human PRV infections in Southeast Asia.

In the present study, we performed sensitive and specific serological assays to diagnose, screen and confirm emerging human PRV infections. The assays allowed for three strains to be identified: the Indonesian/Japanese,^6^ Indonesian/Hong Kong^7, 8^ and Malaysian^2, 3, 4, 5^ strains. The use of these assays provided valuable information on the nature of PRV. In addition, given the recent increase in the number of reported PRV infections in humans^1, 2, 3, 4, 5, 6, 7, 8^ and bats,^2, 9, 10, 11, 12, 13, 14^ these assays will be useful for determining the distribution of PRV.

## MATERIALS AND METHODS

### Recombinant baculoviruses and protein purification

The small segment genes of the Miyazaki-Bali/2007 PRV major outer capsid protein (MOCP) and the cell attachment protein (CAP) genes of the Miyazaki-Bali/2007, HK23629/07 and Melaka PRVs were inserted into recombinant baculoviruses with a histidine-tag gene downstream of the polyhedrin promoter (Ac-His), as previously described.^17, 18^ The Miyazaki-Bali/2007 PRV genes were amplified by PCR using pre-designed primer sets (Supplementary Table S1), while those of the HK23629/07 and Melaka PRVs were chemically synthesized. The GenBank accession numbers of the nucleotide sequences corresponding to these genes are shown in Supplementary Table S1. The amplified DNA was digested with *Bam*HI and then ligated to the *Bam*HI site of the recombinant transfer vector pAcYM1-His^18, 19^ using T4 DNA ligase (Roche, Mannheim, Germany) to generate the recombinant pAcYM1-His constructs. Sf9 insect cells were then co-transfected^20^ with the recombinant pAcYM1-His constructs and linearized baculovirus DNA (BD Biosciences, Franklin Lakes, NJ, USA), according to the manufacturer's instructions, to generate the recombinant baculoviruses.

The recombinant (r) proteins were purified from Tn5 insect cells infected with Ac-His-Miyazaki-Bali/2007 PRV-MOCP, Ac-His Miyazaki-Bali/2007 PRV-CAP, Ac-His HK23629/07 PRV-CAP and Ac-His Melaka PRV-CAP, and then they were solubilized in phosphate-buffered saline (PBS) containing 8 mol/L urea, as previously described.^17, 21^ The expression of each recombinant protein was checked by SDS-PAGE and confirmed by western blot analysis using an anti-His antibody (Qiagen Inc, Valencia, CA, USA).

### Polyclonal antibodies

To generate polyclonal antisera against the Miyazaki-Bali/2007 PRV-MOCP and the CAPs of the Miyazaki-Bali/2007, HK23629/07 and Melaka PRVs, two rabbits each were immunized with the following Ac-His purified proteins as previously described: the Miyazaki-Bali/2007 PRV-rMOCP and the rCAPs of Miyazaki-Bali/2007, HK23629/07 and Melaka PRV.^18, 22^ The protocols of the animal experiments were approved by the Animal Care and Use Committee of the National Institute of Infectious Diseases, Tokyo, Japan (NO. 214078).

## ELISA

A 2-step ELISA approach was established in the present study. As the first step for the screening of anti-PRV antibodies, Miyazaki-Bali/2007 PRV-rMOCP, a protein that is conserved among the different PRV strains, was used as the ELISA antigen. Miyazaki-Bali/2007 PRV-MOCP shares 96.6%–99.4% amino acid homology with the other PRV strains (Supplementary Table S2, Supplementary Figure S1).^2, 3, 4, 5, 6, 7, 8, 10, 11, 12, 13, 14^ In the second step, which was established to identify the PRV strains, the rCAPs of Miyazaki-Bali/2007, HK23629/07 and Melaka PRV served as the ELISA antigens. The CAP is a less conserved protein among the different PRV strains (Supplementary Table S2, Supplementary Figure S2).^2, 3, 4, 5, 6, 7, 8, 9, 10, 11, 12, 13, 14, 15, 16^ The negative antigen was produced by following the same methods as were used to produce the recombinant virus antigens by using Tn5 cells that were infected with the recombinant baculovirus without the polyhedrin gene (Δp).

The standard ELISA protocol was followed^17, 23^ using a predetermined optimal antigen quantity that was produced by diluting the antigen stock to 1:800 with PBS. The detection capacity of the MOCP-based ELISA system was tested by screening rabbit anti-Miyazaki-Bali/2007 PRV rMOCP hyperimmune and preimmunization sera for anti-PRV antibodies. The cross-reactivity between the rabbit polyclonal anti-rCAP sera and the rCAPs of the three PRVs was analyzed using the rCAP-based ELISA. The ΔP antigen was used for each of the MOCP and CAP-based ELISA assays. The optical density at 405 nm (OD_405_) value of ΔP was subtracted from the OD_405_ value obtained for each recombinant protein tested. Anti-His tag antibodies served as controls to confirm that the same quantity of rCAP antigens was used.

### Neutralization test (NT)

Rabbit polyclonal antisera raised against the rCAP of each strain was used to determine the neutralization and cross-neutralization activity against the Miyazaki-Bali/2007 PRV strain as previously described.^15^ Briefly, the serum samples were diluted four-fold from 1:20 to 1:5120 in Dulbecco's modified Eagle's medium (DMEM) containing 2% fetal calf serum. Each 50 μL sample was then mixed with 200 plaque forming units of Miyazaki-Bali/2007 PRV in 50 μL of DMEM, and the mixture was incubated for 1 h at 37 °C for neutralization. After incubation, Vero cell monolayers were inoculated with the mixtures for 1 h. The inoculants were removed, and the cells were cultured for five days with DMEM containing 2% FCS and 1% agarose. The plaques produced by Miyazaki-Bali/2007 PRV were counted under a light microscope. The end-point neutralizing antibody titer was determined by the highest dilution of serum to reduce the plaques by 50% in comparison to the controls (PRNT_50_).

### Immunofluorescence assay

Antigen slides were prepared for an immunofluorescence assay (IFA) using Miyazaki-Bali/2007 PRV-infected 293T cells. The virus-positive cells and mock-infected cells were mixed at a ratio of 1:3, as previously described.^15^ Briefly, rabbit polyclonal antiserum or human serum samples were diluted two-fold with PBS containing 0.05% Tween-20 from 1:10 to 1:2560. 20 μL of each dilution was spotted over each well. The slides were then incubated for 1 h at 37 °C. Following incubation, the slides were washed with PBS three times and then spotted with 20 μL of 1:400 Alexa Fluor 488 goat anti-rabbit or human IgG (Thermo Fisher Scientific, Inc, Waltham, MA, USA) in PBS containing 0.05% Tween-20. The slides were washed again in the manner described above and examined for signals under a fluorescence microscope. The end-point antibody titer was defined as the highest dilution of serum to show a positive signal under the fluorescence microscope.

### Western blotting analysis

The western blotting antigen consisted of Miyazaki-Bali/2007 PRV-infected 293T cells and mock-infected cells. Briefly, Miyazaki-Bali/2007 PRV-infected 293T cells (multiplicity of infection: 3 per cell) or mock-infected cells were cultured for 24 h. The cells were then pelleted, washed with PBS and resuspended in PBS containing 1% Nonidet P-40. Western blotting was performed as previously described^24, 25^ using each of the different rabbit polyclonal antisera produced in this study or in patient serum.

### Validation

The different assays established in the present study were validated to confirm their reactivity and specificity using serum that was previously collected from a Miyazaki-Bali/2007 PRV-infected patient and 46 of the patient's contacts, which included the patient's family members, healthcare providers and local health station staff.^6^ Although the NT against Miyazaki-Bali/2007 PRV had previously been performed, we repeated the NT in the present study to confirm the validity of our assay.^6^ For the detection of anti-PRV antibodies by MOCP-based ELISA (first-step screening), the mean and standard deviation was determined using a total of 18 serum samples from healthy donors. The cutoff value for the assay was defined as the mean plus three standard deviations.

### Ethical statement

The serum samples that were used were collected under informed consent from a Miyazaki-Bali/2007-infected patient and the patient's contacts.^6^ The protocol of this study was approved by the Ethics Committee of the National Institute of Infectious Diseases, Tokyo, Japan (NO. 452).

## RESULTS

### The detection of anti-CAP antibodies

**Figure 1:**
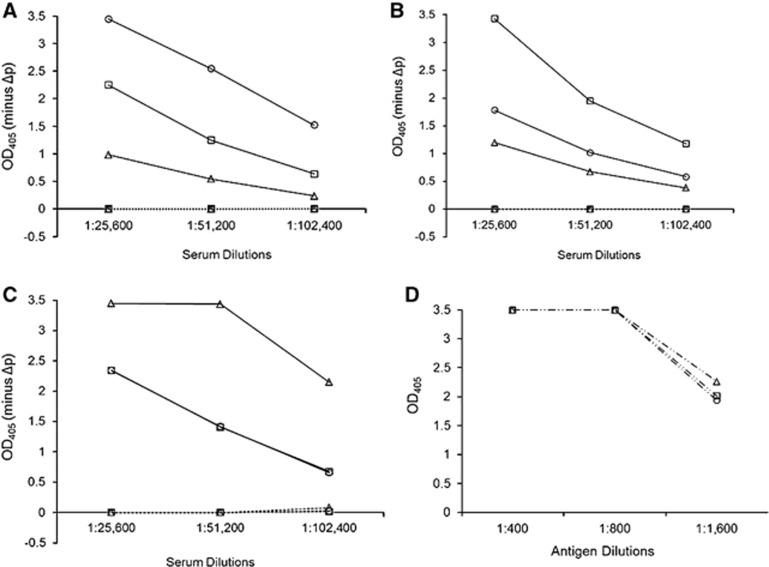
The strain specificity of the rabbit anti-PRV rCAP antibodies against the rCAPs of the different PRVs. IgG ELISA shows the reactivity in the polyclonal antisera raised in rabbits for (**A**) Miyazaki-Bali/2007, (**B**) HK23629/07 and (**C**) Melaka PRV–CAP and (**D**) anti-His tag antibody control (which was used to confirm the antigen quantity) with the rCAPs of Miyazaki-Bali/2007 (○), HK23629/07 (□) and Melaka (Δ) PRVs as the ELISA antigen. ───────, hyperimmune serum; •••••••••••••••••, preimmunization serum; ─ •• ─ •• ─, anti-His tag antibody. OD_405_, optical density at 405 nm against a reference wavelength of 490 nm; Δp, recombinant baculovirus without the polyhedrin gene. The representative results of one rabbit per group are shown. An anti-His tag antibody control was used to confirm that equal quantities of rCAP antigens (800) were used. The *y*-axis represents OD_405_−ΔP. Abbreviations: PRV, *Pteropine orthoreovirus*; rCAP, recombinant cell attachment protein.

As expected, the rabbit anti-Miyazaki-Bali/2007 PRV-MOCP sera showed a positive reaction in the Miyazaki-Bali/2007 PRV MOCP-based ELISA (data not shown). The potential use of the CAP-based ELISA for the differentiation of PRV strains was also observed. Rabbit polyclonal antisera raised against the CAP of a particular strain (for example, Miyazaki-Bali/2007 PRV) showed the highest OD_405_ value to the rCAP antigen of that same strain and, to a lesser extent, cross-reactivity with the other strains (Figures 1A–1C), while the anti-His tag antibody showed the same OD_405_ values for the three rCAP antigens (Figure 1D). The cross-reactivity of the rabbit polyclonal antisera, which were raised against the different PRVs, was also demonstrated by IFA and western blotting. The IFA titers of the rabbit sera raised against Miyazaki-Bali/2007 PRV-rMOCP and rCAP (1:280) were higher than those of the rabbit sera raised against the rCAPs of HK23629/07 and Melaka PRV (both 1:320) (Table 1). All of the rabbit polyclonal sera that were raised in this study were also found to have activity against Miyazaki-Bali/2007 PRV by western blotting (Table 1, Supplementary Figure S3).

On the other hand, the neutralization assays indicated no cross-reactivity between the rabbit anti-CAP sera of the other strains (HK23629/07 and Melaka) against Miyazaki-Bali/2007 PRV (Table 1). The rabbit anti-Miyazaki-Bali/2007 PRV CAP serum showed strong neutralization activity against the Miyazaki-Bali/2007 PRV strain (neutralization titer: 320); in contrast, the anti-Miyazaki-Bali/2007 PRV MOCP antibody had a neutralization titer of 20 (Table 1).

### Validation

The convalescent phase serum of the patient infected with Miyazaki-Bali/2007 PRV showed a positive reaction in the MOCP-based ELISA, while the acute phase serum of the patient did not (Table 2). Similarly, the convalescent phase serum showed a positive reaction in the IFA at a titer of 320, while the acute phase serum was positive at a titer of 10 (Table 2). In addition, a significant increase in the NT score was observed between the acute and the convalescent phases, while all of the NT-negative healthy donor serum samples were found to be negative by the MOCP-based ELISA, IFA and western blotting (Table 2). Similarly, none of the Miyazaki-Bali/2007 PRV infected patient's contacts showed the presence of anti-PRV antibodies by the MOCP-based ELISA, IFA or NT (Table 2). These results indicate the high specificity of the antibody detection assays; however, further studies are needed to evaluate their sensitivity.

**Figure 2:**
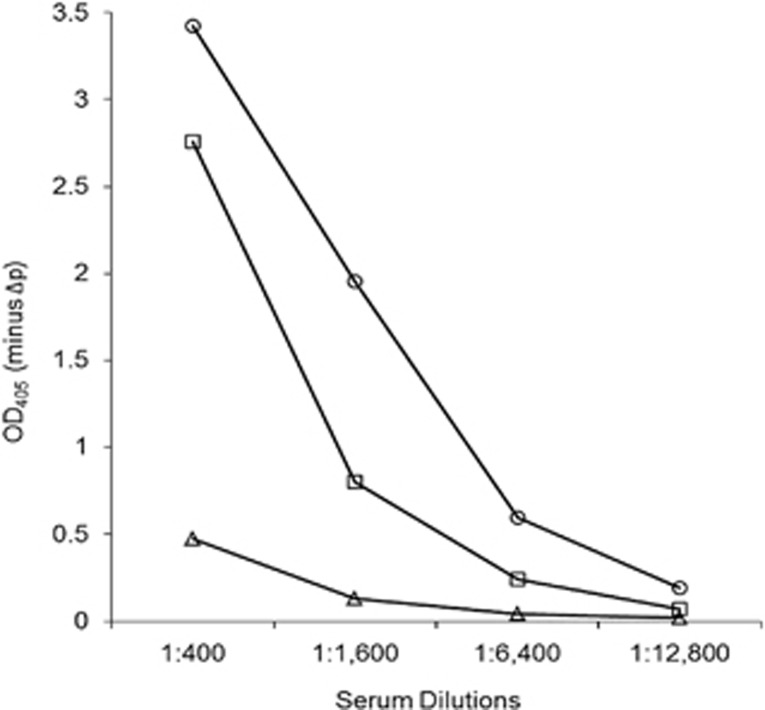
An IgG ELISA using serum from the Miyazaki-Bali/2007 PRV-infected patient in the convalescent phase with the recombinant cell attachment protein of Miyazaki-Bali/2007 (○), HK23629/07 (□) and Melaka (Δ) PRVs as ELISA antigens. OD_405_, optical density at 405 nm against a reference wavelength of 490 nm; Δp, recombinant baculovirus without the polyhedrin gene. The *y*-axis represents OD_405_−ΔP. Abbreviation: PRV, *Pteropine orthoreovirus*.

**Figure 3:**
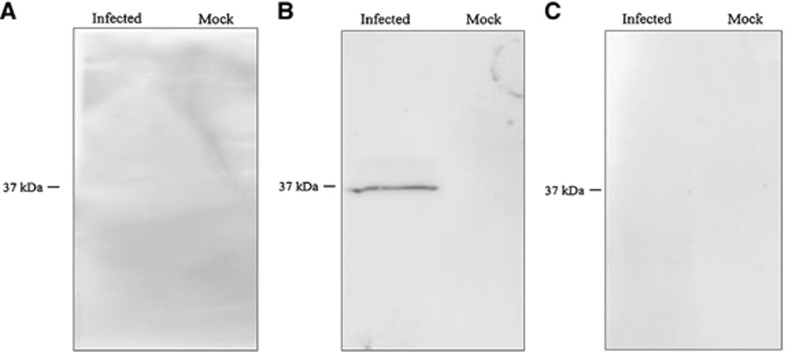
Serologic analysis (western blotting (IgG)) of the serum of the Miyazaki-Bali/2007 PRV-infected patient in the (**A**) acute and (**B**) convalescent phases, as well as the (**C**) healthy donor serum against Miyazaki-Bali/2007 PRV at a primary antibody dilution of 1:1000. Infected: Miyazaki-Bali/2007 PRV-infected 293T cell lysate; mock: mock-infected 293T cell lysate.

The patient's convalescent phase serum showed a stronger reaction against Miyazaki-Bali/2007 PRV-rCAP than against HK23629/07 PRV-rCAP or Melaka PRV-rCAP (Figure 2). The detection of anti-PRV antibodies by western blotting using the convalescent serum of the Miyazaki-Bali/2007 PRV-infected patient and the absence of anti-PRV antibodies in the serum of the patient's contacts further validates these assays (Table 2, Figure 3).

## DISCUSSION

The detection of the anti-PRV antibodies that were validated in this study using the Miyazaki-Bali/2007 PRV MOCP (a protein that is conserved among the different PRV strains), is useful for the screening and diagnosis of PRV infections and possible infections caused by other orthoreovirus species.^2, 6, 14, 26, 27, 28, 29^ The absence of anti-PRV antibodies among the contacts of the Miyazaki-Bali/2007 PRV-infected patient, as shown in this study, suggests that human-to-human transmission of PRV was not likely, despite the development of symptoms among several of the patient's contacts.^6^

The results obtained in the present study suggested that antibodies against different PRVs (even closely related strains) could be distinguished by CAP-based ELISAs. The results of this study suggest that the simultaneous use of the three rCAP-based ELISAs may be useful for the detection of antibodies in human serum to each of the three groups of strains (Miyazaki-Bali/2007, HK46686/09, HK50842/10 and Kampar virus with the rCAP of Miyazaki-Bali/2007 PRV; Sikamat/MYS/2010, Melaka virus and HK23629/07 with the rCAP of Melaka PRV; and HK23629/07 with the rCAP of HK23629/07 PRV, respectively). The identification of the strain of PRV infection is useful in assessing the geographic distribution of infections among closely related strains of this diverse group of viruses. It is possible that PRV variants may exist in bats in Southeast Asia,^2^ due to the marked divergence that is observed, even among closely related orthoreovirus strains.^27, 28^ The circulation of the PRV variants in nature may cause emerging infections in humans. Although this study was limited by the availability of the different viral strains, it demonstrated (by ELISA and IFA), that the rabbit polyclonal antisera raised against the CAP of HK23629/07 and Melaka PRV had lower cross-reactivity with that of Miyazaki-Bali/2007 PRV. The CAP of Miyazaki-Bali/2007 PRV shares a 57.3 and 56.2% homology with the CAP of Melaka and HK23629/07 PRV, respectively.^6, 15^

The CAP of orthoreoviruses has previously been reported to induce the production of neutralizing antibodies during an infection.^27^ In this study, the neutralization assays suggested that the neutralizing antibodies raised against CAP do not offer cross-protection against different PRV strains. Because various PRVs may exist in Southeast Asia, reinfection episodes, with different PRV strains, may possibly occur in humans.^15^

Although PRV infections in humans are generally mild and exhibit a limited capacity for human-to-human transmission, they exist as a diverse group, and their virulence remains unknown. The expanding geographic distribution of reported cases and the possibility of reinfection with different PRV strains suggest the need for close monitoring of PRV infections, especially in Southeast Asia.^2, 6, 8, 15, 27, 28^ The development of an ELISA system using the rMOCP and rCAP of PRV as antigens, as shown in this study allows for the screening, confirmation and strain differentiation of infections with PRV and other orthoreovirus infections in humans. The NT, IFA and western blotting assays can also be used as confirmatory assays for samples that are reactive during ELISA screenings. The use of these assays in the diagnosis and surveillance of PRV infections in humans may contribute to the development of prevention and control strategies.

## Figures and Tables

**Table 1:** Antigenicity and cross-antigenicity among the different rabbit anti-*Pteropine orthoreovirus* (PRV) hyperimmune sera raised in this study with Miyazaki-Bali/2007 PRV^a^

**Serum sample**		**NT**^b^	**IFA**^c^	**WB**^d^
Rabbit anti-Miyazaki-Bali/2007 PRV^e^	rMOCP	20	1280	+
	rCAP	320	1280	+
				
Rabbit anti-HK23629/07 PRV-rCAP^e^		<20	320	+
				
Rabbit anti-Melaka PRV-rCAP^e^		<20	320	+
				
Preimmunization sera^f^		<20	<10	−

Abbreviations: immunofluorescence assay, IFA; Neutralization test, NT; recombinant cell attachment protein, rCAP; recombinant major outer capsid protein, rMOCP; western blotting, WB.

a The representative results of one rabbit per group are shown.

b The neutralization titer was defined as the concentration of serum to reduce the number of plaques produced by Miyazaki-Bali/2007 PRV by 50% in comparison to the serum-free virus controls (PRNT_50_). The NTs were carried out in triplicate.

c The IFA results represent antibody titers against Miyazaki-Bali/2007 PRV-infected 293T cells.

d The results represent the presence (+) or absence (−) of antibodies against Miyazaki-Bali/2007 PRV-infected 293T cell lysate at a primary antibody dilution of 1:1000.

e Similar antibody titers were observed in these rabbit polyclonal antisera against their respective rCAP antigen.

f The titers shown were consistent in all of the preimmunization rabbit serum samples that were used in the assays.

**Table 2:** Serologic assay validation using serum from a patient infected with Miyazaki-Bali/2007 *Pteropine orthoreovirus* (PRV)

**Serum sample**		**ELISA**^a^	**NT**^b^	**IFA**^c^	**WB**^d^
Miyazaki-Bali/2007 PRV-infected patient	Acute phase serum	<100	<20	10	−
	Convalescent phase serum	1600	320	320	+
					
Miyazaki-Bali/2007 PRV-infected patient contacts^e^		<100	<20	<10	−
					
Healthy donors sera^e^		<100	<20	<10	−

Abbreviations: Enzyme-linked immunosorbent assay, ELISA; immunofluorescence assay, IFA; neutralization test, NT; western blotting, WB.

a Small gene segment encoding major outer capsid protein of PRV (MOCP)-based ELISA was used in screening for anti-PRV IgG antibodies. The titers were determined based on the cutoff values established for the IgG ELISA using healthy donor serum samples (mean+3 standard deviations).

b The neutralization titer was defined as the concentration of serum to reduce the number of plaques produced by Miyazaki-Bali/2007 PRV by 50% in comparison to the serum-free virus controls (PRNT_50_). The NTs were carried out in triplicate.

c The IFA results represent antibody titers against Miyazaki-Bali/2007 PRV-infected 293T cells.

d The results represent the presence (+) or absence (−) of IgG antibodies against Miyazaki-Bali/2007 PRV-infected 293T cell lysate at a primary antibody dilution of 1:1000.

e The titers shown were consistent in all contacts and the healthy donor sera used for each assay.
