# Supplementary material for: Serologic assays for the detection and strain identification of Pteropine orthoreovirus
Source: Emerg Microbes Infect. 2016 May 11;5(5):e44–. doi: 10.1038/emi.2016.35 (PMC4893542; doi:10.1038/emi.2016.35)
Supplement: Supplementary Table S1 [file emi201635x1.pdf]

Supplementary Figure S1 The multiple sequence alignment of the small (S) gene segment encoding the major outer capsid protein of different *Pteropine Orthoreovirus* strains from human cases. The conserved regions in the aligned sequence are indicated by black background shading; gray shading denotes the conservation of similar residues. The GenBank accession numbers of the amino acid sequences are indicated in parentheses beside each strain.

```
Miyazaki-Bali/2007 (AB521796)   1 MEVRTPNFHSFVESAYSSYLTRPDTWNPSTLWETGQFVTPDVIRLGNAYCCAQCCGVLYY 60
HK46886/09 (JF803300)           1 ---RTPNFHSFVESAYSSYLTRPDTWNPSTLWETGQFVTPDVIRLGNAYCCAQCCGVLYY 57
HK50842/10 (JF803301)           1 ---RTPNFHSFVESAYSSYLTRPDTWNPSTLWETGQFVTPDVIRLGNAYCCAQCCGVLYY 57
Kampar (EU448337)               1 MEARTPNFHSFVESAYSSYLTRPDTWNPSTLWETGQFVTPDVIRLGNAYCCAQCCGVLYY 60
Sikamat/MYS/2010 (JF811583)     1 MEVRTPNFHSFVESAYSSYLTRPDTWNPSTLWETGQFVTPDVIRLGNAYCCAQCCGVLYY 60
Melaka (EF026046)               1 MEVRTPNFHSFVESAYSSYLTRPDTWNPSTLWETGQFVTPDVIRLGNAYCCAQCCGVLYY 60
HK23629/07 (EU170367)           1 ---RTPNFHSFVESAYSSYLTRPDTWNPSTLWETGQFVTPDVIRLGNAYCCAQCCGVLYY 57

Miyazaki-Bali/2007 (AB521796)  61 GYRPINLYPFPHHRCHQLHRRETSPLLKVVRVGRTTDMVREHYAVWLQSLVDHLSDEKNI 120
HK46886/09 (JF803300)          58 GYRPINLYPFPHHRCHQLHRRETSPLLKVVRVGRTTDMVREHYAVWLQSLVDHLSDEKNI 117
HK50842/10 (JF803301)          58 GYRPINLYPFPHHRCHQLHRRETSPLLKVVRVGRTTDMVREHYAVWLQSLVDHLSDEKNI 117
Kampar (EU448337)              61 GYRPINLYPFPHHRCHQLHRRETSPLLKVVRVGRTTDMVREHYAVWLQSLVDHLSDEKNI 120
Sikamat/MYS/2010 (JF811583)    61 GYRPINLYPFPHHRCHQLHRRETSPLLKVVRVGRTTDMVREHYAVWLQSLVDHLSDEKNI 120
Melaka (EF026046)              61 GYRPINLYPFPHHRCHQLHRRETSPLLKVVRVGRTTDMVREHYAVWLQSLVDHLSDEKNI 120
HK23629/07 (EU170367)          58 GYRPINLYPFPHHRCHQLHRRETSPLLKVVRVGRTTDMVREHYAVWLQSLVDHLSDEKNI 117

Miyazaki-Bali/2007 (AB521796) 121 QDKEPMSSRVASEVLRNDVAISGDFWRGTLDLSPHDSAQSIHDSMDKLSEVAGTHGLLPD 180
HK46886/09 (JF803300)         118 QDKEPMSSRVASEVLRNDVAISGDFWRGTLDLSPHDSAQSIHDSMDKLSEVAGTHGLLPD 177
HK50842/10 (JF803301)         118 QDKEPMSSRVVSEVLRNDVAISGDFWRGTLDLSPHDSAQSIHDSMDKLSEVAGTHGLLPD 177
Kampar (EU448337)             121 QDKEPMSSRVASEVLRNDVAISGDFWRGTLDLSPHDSARSIHDSMDKLSEVAGTHGLLPD 180
Sikamat/MYS/2010 (JF811583)   121 QDKEPMSSRVASEVLRNDVAISGDFWRGTLDLSPHDSAQSIHDSMDKLSEVAGTHGLLPD 180
Melaka (EF026046)             121 QDKEPMSSRVASEVLRNDVAISGDFWRGTLDLSPHDSARSIHDSMDKLSEVAGTHGLLPD 180
HK23629/07 (EU170367)         118 QDNEPMSSRVASEVLRNDVAISGDFWRGTLDLSPHDSAQSIHDSMDKLSEVAGTHGLLPD 177

Miyazaki-Bali/2007 (AB521796) 181 CLLNTATHDHTALRMTAPVLNIYDQLDNARRSVLSATVTQMPKETADAHRLQGHVTKEVA 240
HK46886/09 (JF803300)         178 CLLNTATHDHTALRMTAPVLNIYDQLDNARRSVLSATVTQMPKEAADAHKLQGHVTKEVA 237
HK50842/10 (JF803301)         178 CLLNTATHDHTALRMTAPVLNIYDQLDNARRSVLSATVTQMPKETADAHKLQGHVTKEVA 237
Kampar (EU448337)             181 CLLNVATHDHTALKMTAPVLNIYDQLDNARRSVLSATVTQMPKETADAHKLQGHVTKEVA 240
Sikamat/MYS/2010 (JF811583)   181 CLLNVATHDHMALRMTAPVLNIYDQLDNARRSVLSATVTQMPKETADEHKLQGHVTKEVA 240
Melaka (EF026046)             181 CLLNVATHDHTALKMTAPVLNIYDQLDNARRSVLSATVTQMPKETADAHKLQGHVTKEVA 240
HK23629/07 (EU170367)         178 CLLNTATHDHTALRITAPVLNIYDQLDNARRSVLSATVTQMPKETADAHKLQGHVTKEVA 237

Miyazaki-Bali/2007 (AB521796) 241 AAALLKPEHVIVTPIMAGLPVLARSHQPSLRVGMPLVESTQKASIYRSLPHQLAHGYHSD 300
HK46886/09 (JF803300)         238 AAALLKPEHVIVTPIMAGLPVLARSHQPSLRVGMPLVESTQKASIYRSLPHQLAHGYHSD 297
HK50842/10 (JF803301)         238 AAALLKPEHVIVTPIMAGLPVLARSHQPSLRVGMPLVESTQKASIYRSLPHQLAHGYHSD 297
Kampar (EU448337)             241 AAALLKPEHVIVTPIMAGLPILARSHQPSLRVGMPLVDSTQKASIYRSLPHQLAHGYHSD 300
Sikamat/MYS/2010 (JF811583)   241 AAALLKPEHVIVTPIMAGLPVLARSHQPSLRVGMPLVESTQKASLYRSLPHQLAHGYHSD 300
Melaka (EF026046)             241 AAALLKPEHVIVTPIMAGLPVLARSHQPSLRVGMPLVESTQKASLYRSLPHQLAHGYHSD 300
HK23629/07 (EU170367)         238 AAALLKPEHVIVTPIMAGLPVLARSHQPSLRVGMPLVESTQKASIYRSLPHQLAHGYHSD 297

Miyazaki-Bali/2007 (AB521796) 301 TFRTMFGLSANEAESRLIGHHRTMLCTLPAIETSSTAVARATASPLSIVSSDKVTMHECS 360
HK46886/09 (JF803300)         298 TFRTMFGLSANEAESRLIGHHRTMLCTLPAIETSSTAVARATASPLSIVSSDKVTMHECS 357
HK50842/10 (JF803301)         298 TFRTMFGLSANEAESRLIGHHRTMLCTLPAIETSSTAVARATTSPLSIVSSDKVTMHECS 357
Kampar (EU448337)             301 TFRTMFGLSANEAESRLIGHHRTMLCTLPAIETPSTATARVATSPLSIVSSDKVTMHECS 360
Sikamat/MYS/2010 (JF811583)   301 TFRTMFGLSANEAESRLIGHHRTMLCTLPAIETSSTAVARATTSPLSIVSSDKVTMHECS 360
Melaka (EF026046)             301 TFRTMFGLSANEAESRLIGHHRTMLCTLPAIETSPTAVARATTSPLSIVSSDKVTMHECS 360
HK23629/07 (EU170367)         298 TFRTMFGLSANEAESRLIGHHRTMLCTLPAIETSSTAVARATASPLSIVSSDKVTMHECS 357

Miyazaki-Bali/2007 (AB521796) 361 G                                                            361
HK46886/09 (JF803300)         358 G                                                            358
HK50842/10 (JF803301)         358 G                                                            358
Kampar (EU448337)             361 G                                                            361
Sikamat/MYS/2010 (JF811583)   361 G                                                            361
Melaka (EF026046)             361 G                                                            361
HK23629/07 (EU170367)         358 G                                                            358
```
