# Supplementary material for: Serologic assays for the detection and strain identification of Pteropine orthoreovirus
Source: Emerg Microbes Infect. 2016 May 11;5(5):e44–. doi: 10.1038/emi.2016.35 (PMC4893542; doi:10.1038/emi.2016.35)
Supplement: Supplementary Table S2 [file emi201635x2.pdf]

Supplementary Figure S2 The multiple sequence alignment of the cell attachment proteins of different *Pteropine Orthoreovirus* strains from human cases. The conserved regions in the aligned sequence are indicated by black background shading; gray shading denotes the conservation of similar residues. The GenBank accession numbers of the amino acid sequences are indicated in parentheses beside each strain.

```
Miyazaki-Bali/2007 (AB521793)   1 -MDPMSSSTTLHSLSPPQRREVIALILTMTQSTSASRSDLSALRSDLNSMSVALTNLTGI 59
HK46886/09 (JF803294)           1 -MDPMSSSTTLHSLSPPQRREVIALILTMTQSTSASRSDLSALRSDLNSMSVALTNLTGI 59
HK50842/10 (JF803295)           1 -MDPMSSSTTLHSLSPPQRREVIALILTMTQSTSASRSDLSALRSDLNSMSVALTNLTGI 59
Kampar (EU448334)               1 -MDPMLSSTTLHSLSPPQRREVIALILTMTQSTSASRSDLSAVRSDLNSMSVALTNLTGI 59
Sikamat/MYS/2010 (JF811580)     1 MTDLMSSFTIHHSLNPQQRREVIALIMTMTQSISASRSDLSALRDELTSLRTKVADVSSQ 60
Melaka (EF026043)               1 ----MSSFTIHHSLNPQQRREVIALIMTMTQSISASRSDLSALRDELASLRTKVTDVSSQ 56
HK23629/07 (EU165526)           1 -MDPTLLSTIHHSLSPPQRREVIALIMTMTQSISASRSDLSATRAELTSLLTRVDSLTSQ 59

Miyazaki-Bali/2007 (AB521793)  60 VSKHTTDISSITSDLSSLAGKLDSQLSELDGLKTSISSNLTNLQSLSSRVSSVSNDLAAV 119
HK46886/09 (JF803294)          60 VSKHTTDISSITSDLSSLAGKLDSQLSELDGLKTSISSNLTNLQSLSSRVSSVSNDLAAV 119
HK50842/10 (JF803295)          60 VSKHTTDISSITSDLSSLAGKLDSQLSELDGLKTSISSNLTNLQSLSSRVSSVSNDLAAV 119
Kampar (EU448334)              60 VSKHTTDISSITSDLSSLTGKLDSQLSELDELKTNVSSNLTDLQSLSSRVSSVSNDLAAV 119
Sikamat/MYS/2010 (JF811580)    61 LASTSSTITQLSTQCSALSAELTSYSNTLSTLSSTIASHTDKLSELSTSLTSASSRLDAT 120
Melaka (EF026043)              57 LALASSTITQLSTQCSALSAELASYSDTLSTLSSTIASHTDKLSELSTSLTSTSSRLDAT 116
HK23629/07 (EU165526)          60 ITTMSSTLSQLSDQFSSLSSSVTSNTNNLDSLSKDVQSHSVMLSEFSSSLNNISTTLTDT 119

Miyazaki-Bali/2007 (AB521793) 120 SSSLEQLSGKLNVTTTDVTNLQTSVSTMAAQLSALDSKLNDTAQRLPQQVSAPLVIDNGS 179
HK46886/09 (JF803294)         120 SSSLEQLSGKLNVTTTDVTNLQTSVSTMAAQLSALDSKLNDTAQRLPQQVSAPLVIDNGS 179
HK50842/10 (JF803295)         120 SSSLEQLSGKLNVTTTDVTNLQTSVSTMAAQLSALDSKLNDTAQRLPQQVSAPLVIDNGS 179
Kampar (EU448334)             120 NSSLEQLSGKLNVTATDVTNLQNSVSTMAAQLSALDSKLNDTAQRLPQQVSAPLVIDNGS 179
Sikamat/MYS/2010 (JF811580)   121 SDSVTQLSSDYASLRTDVTNLKSSLSTLAAQVNSLETKLNDTNQTVPKQVLSPLAINDGT 180
Melaka (EF026043)             117 ADSVTQLSSDYASLRTDVTNLKSSLSTLAAQVNSLETKLNDTTQTVPKQVLSPLAINDGT 176
HK23629/07 (EU165526)         120 SNSVTRLSGQYAMLETDVANSKSSLAIMASQLKSLESTLNDATQSIPRQVTAPLSISNGS 179

Miyazaki-Bali/2007 (AB521793) 180 LALQMNPRFCRDELGLNSYGSQTLLQTFNANVVTNVTGTNLATTIIVHSRGSYSTFNLTS 239
HK46886/09 (JF803294)         180 LALQMNPRFCRDELGLNSYGSQTLLQTFNANVVTNVTGTNLATTIIVHSRGSYSTFNLTS 239
HK50842/10 (JF803295)         180 LALQMNPRFCRDELGLNSYGSQTLLQTFNANVVTNVTGTNLATTIIVHSRGSYSTFNLTS 239
Kampar (EU448334)             180 LSLQMNPRFCRDDLGLNSYGSQTLLQTFNANVVTNVTGTNLATTIIVHSRGSYSTFNLTS 239
Sikamat/MYS/2010 (JF811580)   181 LTLNMNPRFCRDSAGLASYSSQTLLQTFSANLASSIPDTNLATTIIVHSHGSVSTFNLTS 240
Melaka (EF026043)             177 LTLNMNPRFCRDSAGLASYSSQTLLQTFSANLASSIPNTNLATTIIVHSHGSVSTFNLTS 236
HK23629/07 (EU165526)         180 LTLAMNPRFCRSDAGLSSYSSQTLLQTFSANLTSSLSGTNLATTIIVHSHGSASTFNLTS 239

Miyazaki-Bali/2007 (AB521793) 240 QHAFTPSSTDTQLKLDVRGFKPVPSDWSVLLAKPAFQASDFLGYAWAKCNSVWSPISLIG 299
HK46886/09 (JF803294)         240 QHAFTPSSTDTQLKLDVRGFKPVPSDWSVLLAKPAFQASDFLGYAWAKCNSVWSPISLIG 299
HK50842/10 (JF803295)         240 QHAFTPSSTDTQLKLDVRGFKPVPSDWSVLLAKPAFQASDFLGYAWAKCNSVWFPISLIG 299
Kampar (EU448334)             240 QHAFTPSSTDTRLRLNVREFKPIPSDWSVLLAKPAFQASDFLGYAWAKCNGVWSPISLIG 299
Sikamat/MYS/2010 (JF811580)   241 QHAFEPNAAKTQLTLDIRQFQPTPTDWSVLLAQPAFQASDFLGYAWASVAGIWAPITLVG 300
Melaka (EF026043)             237 QHAFEPNAAKTQLTLDIRQFQPTPTDWSVLLAQPAFQASDFLGYAWASVAGIWAPITLVG 296
HK23629/07 (EU165526)         240 NHAFTPTGEKTQLTLDVRTLQPIPTDWSVLLAQPAFQSSDFLGYAWASISGIWSPITLVG 299

Miyazaki-Bali/2007 (AB521793) 300 RVGDDPKVITLHLGTSTNIRIESLVLTFSIDT                             331
HK46886/09 (JF803294)         300 RVGDDPKVITLHLGTSTNIRIESLV-------                             324
HK50842/10 (JF803295)         300 RVGDNPKVITLHLGTSTNIRIESLV-------                             324
Kampar (EU448334)             300 RVGDDPKVITLHLGTSIDTRIESLVLTFSIDT                             331
Sikamat/MYS/2010 (JF811580)   301 RVDSNPKVITLQLGTTSIDRITGLVLTFSIDT                             332
Melaka (EF026043)             297 RVDSNPKVITLQLGTPSMDRITGLVLTFSIDT                             328
HK23629/07 (EU165526)         300 RVTSDPKVINLYLGTTPNARVTGLV-------                             324
```
