# Supplementary material for: Serologic assays for the detection and strain identification of Pteropine orthoreovirus
Source: Emerg Microbes Infect. 2016 May 11;5(5):e44–. doi: 10.1038/emi.2016.35 (PMC4893542; doi:10.1038/emi.2016.35)
Supplement: Supplementary Figure S1 [file emi201635x3.pdf]

Supplementary Figure S3 Serologic analysis (Western blotting) of polyclonal antisera raised in 2 rabbits each (Rabbit 1, Rabbit 2) - namely (**A**) rabbit anti-Miyazaki-Bali/2007 PRV – recombinant (r) major outer capsid protein (MOCP) antibody, rabbit anti-rCell attachment protein (CAP) of (**B**) Miyazaki-Bali/2007, (**C**) HK23629/07 and (D) Melaka PRV antibody, at a primary antibody dilution of 1:1000 against Miyazaki-Bali/2007 PRV. The molecular mass of the Miyazaki-Bali/2007 PRV small (S) gene segment encoding MOCP was approximately 39.5 kilodaltons (kDa), while that of CAP was approximately 36.5 kDa. Inf., Miyazaki-Bali/2007 PRV-infected 293T cell lysate; Mock, mock-infected 293T cell lysate; +, anti-PRV IgG-positive; -, anti-PRV IgG-negative.

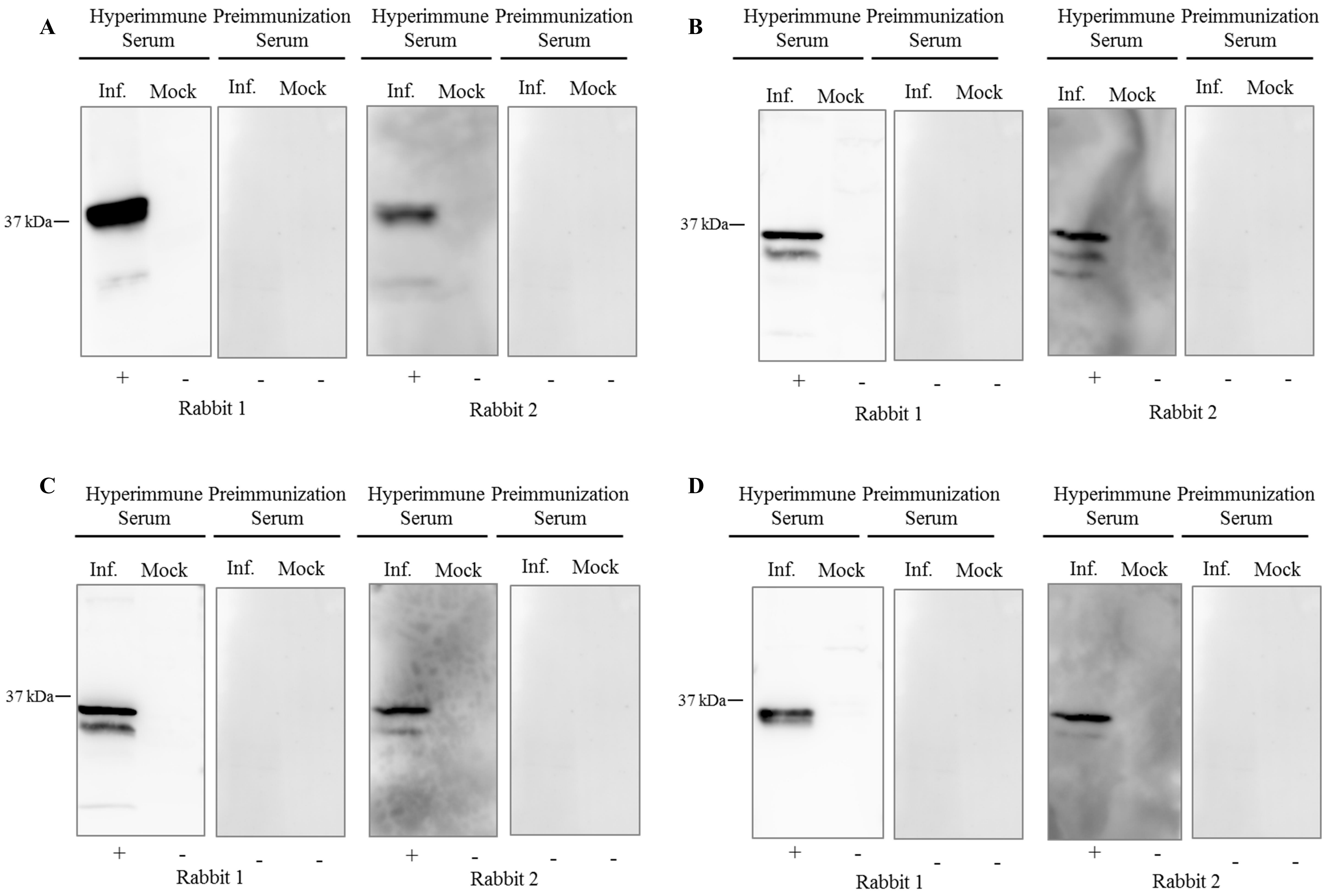
A
B
C
D
Hyperimmune Serum
Preimmunization Serum
Inf.
Mock
37 kDa
+
-
Rabbit 1
Rabbit 2
