# Supplementary material for: Serologic assays for the detection and strain identification of Pteropine orthoreovirus
Source: Emerg Microbes Infect. 2016 May 11;5(5):e44–. doi: 10.1038/emi.2016.35 (PMC4893542; doi:10.1038/emi.2016.35)
Supplement: Supplementary Figure S2 [file emi201635x4.pdf]

Supplementary Table S1 The Genbank accession numbers of the genes of selected PRV strains, and the primers used for the amplification of the cDNA in the present study[a]

PRV Strain	Gene[b]	Genbank accession number	Primer[c]	
			Sequence, 5'to 3'	Polarity
Miyazaki-Bali/2007	MOCP		ACA<u>GGATCC</u>ACCATGGAGGTGAGAACACCCAAC	Forward
			ACA<u>GGATCC</u>CCTGAGCACTCATGCATTGTG	Reverse
	CAP		ACA<u>GGATCC</u>ACCATGGACCCCATGTCGTCATCCAC	Forward
			ACA<u>GGATCC</u>CGTGTCGATAGAGAAGGTGAG	Reverse
HK23629/07	CAP	EU165526		
Melaka	CAP	EF026043		

[a] cDNA clones of Miyazaki-Bali/2007 PRV were amplified by PCR; HK23629/07 and Melaka PRV were chemically synthesized

[b] MOCP, major outer capsid protein; CAP, cell attachment protein.

[c] the *Bam*HI site is underlined.
