# Supplementary material for: Serologic assays for the detection and strain identification of Pteropine orthoreovirus
Source: Emerg Microbes Infect. 2016 May 11;5(5):e44–. doi: 10.1038/emi.2016.35 (PMC4893542; doi:10.1038/emi.2016.35)
Supplement: Supplementary Figure S3 [file emi201635x5.pdf]

Supplementary Table S2 Homology (%) in the amino acid sequences of the small (S) gene segment encoding the major outer capsid protein (columns to the right) and cell attachment protein (column below) of the different *Pteropine Orthoreovirus* (PRV) strains from human cases[a]

		AB521796 Miyazaki-Bali/2007	JF803300 HK46686/09	JF803301 HK50842/10	EU448337 Kampar	JF811583 Sikamat/ MYS/2010	EF026046 Melaka	EU170367 HK23629/07
AB521793	Miyazaki-Bali/2007	-	99.4	99.2	96.7	98.3	98.1	99.2
JF803294	HK46686/09	100.0	-	99.2	96.9	98.3	98.0	99.2
JF803295	HK50842/10	99.4	99.4	-	97.2	98.6	98.3	98.9
EU448334	Kampar	93.7	93.5	92.9	-	96.7	97.5	96.6
JF811580	Sikamat/ MYS/2010	57.1	56.2	56.5	55.6	-	98.6	98.0
EF026043	Melaka	57.3	56.4	56.7	56.1	96.3	-	97.8

EU165526	HK23629/07	56.2	56.2	55.6	56.5	67.3	66.4	-

[a] The amino acid sequences used for the major outer capsid protein (MOCP) and cell attachment protein (CAP) were taken from GenBank. The GenBank accession numbers are indicated in the row above or in the column to the left, respectively, for the MOCP and CAP of each PRV strain.
